# Cellular dynamics of distinct skeletal cells and the development of osteosarcoma

**DOI:** 10.3389/fendo.2023.1181204

**Published:** 2023-05-09

**Authors:** Shohei Otani, Mizuho Ohnuma, Kosei Ito, Yuki Matsushita

**Affiliations:** ^1^ Department of Molecular Bone Biology, Nagasaki University Graduate School of Biomedical Sciences, Nagasaki, Japan; ^2^ Department of Cell Biology, Nagasaki University Graduate School of Biomedical Sciences, Nagasaki, Japan; ^3^ Department of Clinical Oral Oncology, Nagasaki University Graduate School of Biomedical Sciences, Nagasaki, Japan

**Keywords:** osteosarcoma (OS), skeletal stem cells (SSCs), endosteal stem cells, cancer initiating cells, skeletal stem and progenitor cells (SSPCs), bone marrow mesenchymal stem/stromal cells (BM-MSCs), single-cell RNA sequencing (scRNA-seq), lineage-tracing

## Abstract

Bone contributes to the maintenance of vital biological activities. At the cellular level, multiple types of skeletal cells, including skeletal stem and progenitor cells (SSPCs), osteoblasts, chondrocytes, marrow stromal cells, and adipocytes, orchestrate skeletal events such as development, aging, regeneration, and tumorigenesis. Osteosarcoma (OS) is a primary malignant tumor and the main form of bone cancer. Although it has been proposed that the cellular origins of OS are in osteogenesis-related skeletal lineage cells with cancer suppressor gene mutations, its origins have not yet been fully elucidated because of a poor understanding of whole skeletal cell diversity and dynamics. Over the past decade, the advent and development of single-cell RNA sequencing analyses and mouse lineage-tracing approaches have revealed the diversity of skeletal stem and its lineage cells. Skeletal stem cells (SSCs) in the bone marrow endoskeletal region have now been found to efficiently generate OS and to be robust cells of origin under *p53* deletion conditions. The identification of SSCs may lead to a more limited redefinition of bone marrow mesenchymal stem/stromal cells (BM-MSCs), and this population has been thought to contain cells from which OS originates. In this mini-review, we discuss the cellular diversity and dynamics of multiple skeletal cell types and the origin of OS in the native *in vivo* environment in mice. We also discuss future challenges in the study of skeletal cells and OS.

## Introduction

Bone is an important organ that significantly contributes to the maintenance of vital biological activities. It forms the skeleton and helps provide locomotion. In addition, it is the main site of hematopoiesis in the adult body (1). At the cellular level, bone marrow is composed of skeletal cells, hematopoietic cells, and vascular cells. These cells form a functional bone marrow niche by interacting with each other (2–9). Significant progress has been made in researching skeletal and hematopoietic stem cells (SSCs and HSCs, respectively). Both SSCs and HSCs are somatic stem cell types that are found in various body tissues and are involved in their growth, regeneration, and homeostasis. By definition, somatic stem cells are self-renewing and multipotent (10, 11). SSCs are bone tissue-specific mesenchymal stem cells (MSCs). MSCs have been identified in various tissues, including bone, bone marrow, fat, umbilical cord, placenta, synovium, dental pulp, and other tissues (12, 13). Currently, it has been elucidated that SSCs are spatiotemporally located at a part of growth plate cartilage, periosteum, and bone marrow in long bone (14–18). In particular, SSCs have been characterized as self-renewing and having the potential to differentiate into osteoblasts, chondrocytes, and adipocytes. These characteristics were determined using *ex vivo* cell culture experiments, which are considered the gold standard for this type of research (19, 20). Since SSCs were first identified in the 1960s (21), multiple studies have examined skeletal stem and progenitor cells (SSPCs) and their cell lineages, including differentiated chondrocytes, osteoblasts, and marrow adipocytes (1, 22–25). In the past decade, the characteristics of skeletal cells have been better elucidated by using fluorescence-activated cell sorting (FACS)-based isolation techniques based on the use of appropriate cell surface markers. These techniques have been validated in mice (26–28) and humans (29–31). In addition, an *in vivo* lineage-tracing approach using cell type-specific constitutively active *cre* or tamoxifen-inducible *creER* genetic mice with fluorescent reporter strains has been very useful (1, 22, 23, 32–34). Recently, single-cell RNA sequencing (scRNA-seq) has revealed the heterogeneity of skeletal cells at the single-cell level, and this technique has also accelerated research in this field (35). Moreover, a combination of these methods has improved our understanding of skeletal biology.

Osteosarcoma (OS) is the most common malignant bone tumor in children and adolescents. The most favorable sites for OS to develop are in the femur, tibia, and humerus (36). According to the WHO classification of bone tumors, OS can be divided into various subtypes, including conventional (osteoblastic, chondroblastic, and fibroblastic), telangiectatic, small-cell, low-grade central, parosteal, periosteal, high-grade surface, and secondary types (37). Thus, the pathological nature of OS varies, and patient samples in which complete transformation has occurred are generally not suitable for elucidating the mechanisms of OS development. This highlights the need for establishing experimental animal models that faithfully reproduce the development of OS in humans. Above all, studies that make full use of well-constructed mouse models to identify the cells of origin for OS will be indispensable.

A comprehensive understanding of the relationship between skeletal cells and OS is essential for investigating the dynamics and mechanisms of osteosarcomagenesis. Mouse genetic approaches are helpful for discovering the cells of origin and cellular dynamics of OS. In this mini-review, we discuss the progress of skeletal cell research and OS research over the past decade in terms of OS initiation from skeletal cells, as well as future directions and issues.

## Novel techniques to elucidate the skeletal systems in the past decade

In the past decade, significant progress has been made in bone research due to technological improvements and new developments. In particular, FACS-based cell isolation using cell surface markers (26, 27), an *in vivo* lineage-tracing approach using cell-type-specific constitutively active *cre* or tamoxifen-inducible *creER* genetic mice (32, 38, 39), and current scRNA-seq analyses (35) have all been applied to study the diversity and the dynamics of skeletal cells. Furthermore, all these techniques have been well-developed and now exist as robust technologies. Among these, mouse *in vivo* lineage-tracing analysis has been commonly used to investigate spatiotemporally specific cell fates and the functions of skeletal cell subpopulations. This approach uses the *cre*-*loxp* system (32). Crossing transgenic mice that express *cre* recombinase in specific target cells expressing a gene-specific promoter region with knock-in mice that have a *loxp*-*stop*-*loxp* site and a subsequent artificial fluorescent reporter gene in the *Rosa26* locus (40–42) results in offspring with target cells that express fluorescent protein when the gene of interest is expressed. Importantly, after *cre* recombination occurs, the target cells will continue to express the fluorescent protein even after proliferation or differentiation and will not stop unless they undergo apoptosis. Constitutively active *cre* systems are useful in cases in which gene mutations always occur during the initial stages of embryogenesis. All cells with the gene mutations can be fluorescently labeled after *cre* recombination occurs. However, constitutively active *cre* systems are not useful for determining the precise cell lineage of target cells because recombination is induced whenever the promoter is activated and temporal factors cannot be controlled. To avoid these problems, the *creER* system can be used, and the recombination will only be induced after tamoxifen administration (38, 39).

Technology based on RNA-seq is widely used for the comprehensive analysis of gene expression in cell populations. Previous bulk RNA-seq methods only quantified the average gene expression of target cell populations. However, scRNA-seq analysis can be used to study gene expression at the single-cell level. These analyses have revealed cellular heterogeneity, as well as distinct molecular signatures in individual cells. The use of scRNA-seq analysis has rapidly progressed over the past decade (43, 44). Recently, various single-cell-based analyses have been developed, including assays for transposase-accessible chromatin (ATAC)-seq, combined multiome seq (mRNA + ATAC), cellular indexing of transcriptomes and epitopes by sequencing (CITE-seq, mRNA + protein), and single-cell spatial transcriptome analysis (45–49). The combination of *in vivo* lineage-tracing and single-cell sequencing has provided new insights into the diversity of skeletal cells and dynamics of distinct skeletal cells (50).

## ScRNA-seq reveals cellular diversity in the skeletal system

The skeleton is composed of many types of skeletal cells, including osteoblasts, osteocytes, bone marrow stromal cells, chondrocytes, and periosteal cells. However, the large number of hematopoietic and vascular cells in the bone marrow can make it difficult to find bone marrow skeletal cells. Bone marrow stromal cells are vaguely defined as cells that are located between the outer surfaces of the marrow blood vessels and the bone surfaces that encase the hematopoietic space and tissue (51). In addition, a small percentage of skeletal cells can be isolated from whole bone marrow (16, 52). Therefore, the diversity of skeletal cells in bone marrow has not been fully elucidated.

The landscape of skeletal cell populations has been revealed by scRNA-seq studies. Multiple studies have confirmed the heterogeneity of skeletal cells in bone (35, 53–61). In many studies, scRNA-seq analyses are performed in combination with cell surface marker- and/or mouse cell type-specific fluorescence reporter-based FACS. The heterogeneity of mouse bone marrow stromal cells was revealed using these methods. An scRNA-seq analysis of FACS-isolated non-hematopoietic bone marrow cells revealed many skeletal clusters, which included leptin receptor (LepR)^+^ reticular cells, osteoblast lineage cells, pericytes, and fibroblasts (59). These clusters can be further divided into sub-populations. The heterogeneity of LepR^+^ bone marrow perivascular lineage cells and alpha-1 type I collagen (2.3kb Col1a1)^+^ osteoblast lineage cell populations were shown in detail using *Lepr-cre* and *Col1a1(2.3kb)-creER* mice at a steady state. *Lepr-cre* ^+^ cells can be divided into four clusters, including adipogenic and osteogenic populations, whereas *Col1a1(2.3kb)-creER* ^+^ cells can be divided into three clusters, including mature and immature osteoblasts (59). C-X-C motif chemokine 12 (CXCL12, also known as stromal cell-derived factor 1 [SDF1]) and LepR are co-expressed in bone marrow reticular cells (16), and CXCL12-abundant reticular (CAR) cells and LepR^+^ cells mostly overlap. The results of scRNA-seq analyses using Cxcl12-GFP^+^ cells from *Cxcl12* ^GFP/+^ mice or FACS-isolated non-hematopoietic mouse cells have shown that these CAR cells form heterogeneous populations, including adipogenic (Adipo-CAR cells) and osteogenic (Osteo-CAR cells) populations (60–62), as *Lepr-cre* ^+^ cells do. Currently, the diversity of all skeletal cells, which can be positively selected using *Col2a1-cre* (53) or *Prrx1-cre* in young and old mice, has been revealed, and these cells cluster into major differentiated cell types. These include chondrocytes, osteoblasts, stromal/adipocytes, and their transitional populations (18). Multiple studies have identified unique clusters, and their dynamics and molecular mechanisms have been computationally inferred. Trajectory analyses using pseudo-time and RNA velocity can also predict skeletal cell lineages (63–66). However, the results of these analyses are only predictions that depend heavily on bioinformatics. Validation analyses will be required to investigate the lineage and molecular mechanisms of gene expression in distinct cell types.

## *In vivo* lineage-tracing reveals cellular dynamics in the skeletal system

**Figure 1:**
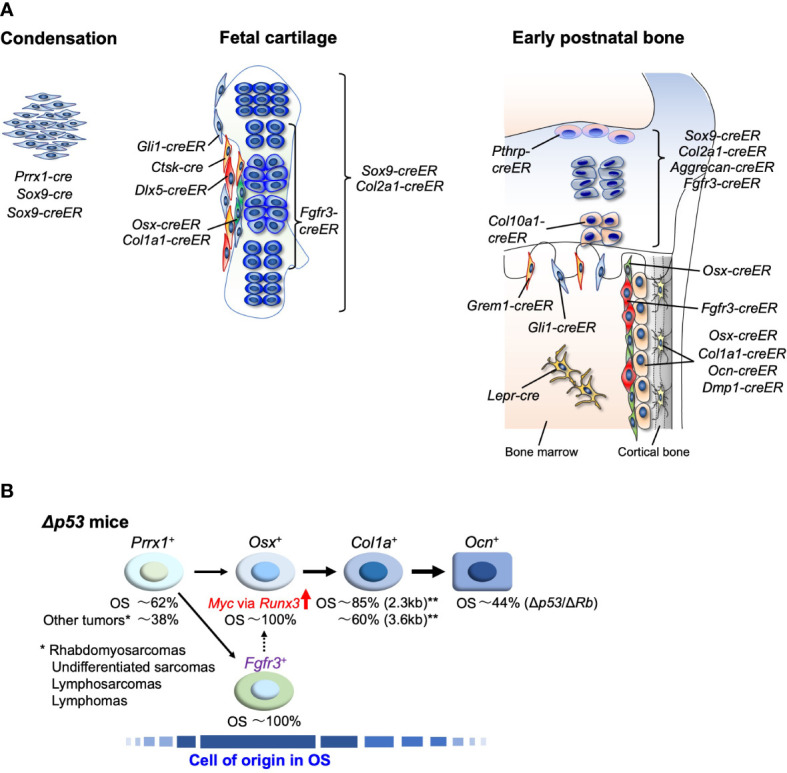
**(A)** Endochondral bone development and cell type-specific *cre* and *creER* lines, During the development, SSPCs contribute to bone formation. These SSPCs and their differentiated cells are targeted by multiple lines. Lineage-tracing approaches reveal cellular dynamics of distinct skeletal cells. **(B)** Approach to the identification of cell-of-origin in OS using *p53-*targeting mice. OS incidence in each cell linage-specific *p53*-targeting mouse line listed in **Table 1** is shown in %. ** show the results of studies using two different length promoters of *Col1a1-cre*.

Lineage-tracing approaches have been widely used to track the fates of skeletal cells. Multiple *cre* or *creER* lines have been used to reveal the cellular dynamics involved (**Figure 1A**). Spatiotemporally specific line choices were also used to precisely determine skeletal cell dynamics.

In fetal long bone development, undifferentiated limb bud mesenchyme marked by *Prrx1-cre* gives rise to *Sox9*-expressing mesenchymal condensations at the first step (90, 91). *Prrx1-cre* lineage cells differentiate into all skeletal cells, and the condensations with *Sox9-cre* or *Sox9-creER* provide most groups of skeletal cells as osteo-chondro progenitors (92, 93). During the next condensation step, the cartilage template and surrounding perichondrium are formed. The *Col2a1-cre* ^+^ or *Col2a1-creER* ^+^ cells appear in both the cartilage template and perichondrium at this stage, and these cells differentiate into postnatal chondrocytes, osteoblasts, stromal cells, marrow adipocytes, pericytes, and periosteal cells (53, 94). *Gli1-creER* predominantly marks perichondrial cells and a few chondrocytes at this stage. These cells give rise to multiple cell types associated with the skeleton (95). Hypertrophic chondrocytes marked by *Col10a1-cre* or *Col10a1-creER* differentiate into postnatal osteoblasts and marrow stromal cells without perichondrial or periosteal cells (96, 97). *Fgfr3-creER* ^+^ cells in the central area of the cartilage template predominantly become postnatal growth plate chondrocytes and metaphyseal skeletal cells, but not to the diaphyseal skeleton (98). In contrast, the outer layer of perichondrial cells marked by *Dlx5-creER* become postnatal diaphyseal osteoblasts, periosteal cells, and stromal cells with adipogenic properties. Interestingly, the inner layers of osteogenic perichondrial cells marked by *Osterix (Osx)-creER* or *Col1a1-creER* transiently become skeletal cells in the bone and bone marrow at the neonatal stage but they disappear from the bone at the postnatal stage (98–100). The *Cathepsin K (Ctsk)-cre* marker is present in the perichondrial area during the embryonic stage and cells with this marker differentiate into the periosteal cells (15).

In the postnatal stage, SSPCs and differentiated cell dynamics can be studied using lineage-tracing analyses. Cell type-specific SSCs in the young stage have been reported in the bones, including *Pthrp-creER* ^+^ growth plate stem cells in the early postnatal resting zone (14) and *Ctsk-cre* ^+^ early postnatal periosteal stem cells (15, 101), whereas *Lepr-cre* ^+^ and *Ebf3-creER* ^+^ CAR cells in the bone marrow stroma behave like SSCs in the adult stage (16, 102). Recently, it was discovered that endosteal stromal cells marked by *Fgfr3-creER* in bone marrow in the young stage behave like SSCs, and they have been named endosteal stem cells (18). These endosteal stem cells differentiate into osteoblasts, CXCL12^+^LepR^+^ reticular cells, and their lineage cells under physiological conditions, and become all skeletal cells under *in vivo* transplantation conditions. Multiple cell lines target SSPCs during postnatal development. Growth plate chondrocytes, marked by *Sox9-creER*, *Aggrecan-creER*, *Col2a1-creER*, and *Col10a1-creER*, become osteoblasts and CXCL12^+^LepR^+^ reticular cells (94, 96, 97). Importantly, these lines predominantly mark growth plate chondrocytes, but they also mark skeletal cells in the metaphyseal area after a short chase of tamoxifen injection. In addition, *Gli1-creER^+^* cells residing in the growth plate and immediately beneath the growth plate are essential for cancellous bone formation as metaphyseal mesenchymal progenitors (95). *Grem1-creER^+^* cells, which are found in the marrow space adjacent to the growth plate and trabecular bone, differentiate into chondrocytes, osteoblasts, and stromal cells (103).

To address osteoblast lineages, osteoblast differentiation-related genes have been applied as *cre* or *creER* driver genes. Osx is an essential transcription factor expressed in osteoblasts and osteoblast precursor cells during the postnatal stage (104, 105). *Osx-cre* marks the cortical and trabecular osteoblasts, periosteal cells, and reticular and perivascular stromal cells (106–108). On the other hand, *Osx-creER* shows intriguing cell dynamics. *Osx-creER^+^* cells behave as skeletal progenitors in the early postnatal stage; they mark osteoblasts, osteocytes, and preosteoblast-like cells in the endosteal space overlaying osteoblasts on the bone surface, and they differentiate into osteoblast lineage cells and stromal cells adjacent to blood vessels in the marrow cavity. However, in adults, *Osx-creER*-marked cells become only osteoblast lineage cells (100). Osteocalcin (OCN) and dentin matrix protein 1 (DMP1) are expressed in osteoblasts and osteocytes during later stages of osteogenesis. *Ocn-cre* and *Dmp1-cre* are commonly used to target osteoblast lineage cells (109, 110). Several studies have revealed that these *cre* lines mark broader stromal cell populations in the bone marrow, as well as in osteoblast lineage cells (111, 112), although *Ocn-creER* and *Dmp1-creER* specifically mark osteoblast lineage cells (113, 114). *Col1a1-cre* targets osteoblast lineage and periosteal cells (115, 116), whereas *Col1a1-creER* mainly marks osteoblasts and osteocytes (117). Several *cre* and *creER* lines have been described and may be used for experimental purposes.

## Cells of origin in osteosarcoma

MSCs are a rare cell population, but they are present in many tissues and serve as a source of mesenchymal progenitor cells (118). Growing evidence suggests that bone marrow (BM)-MSCs (a.k.a. BM-SSCs) may contain sarcoma- or tumor-forming cells, and they have attracted attention in OS research as a possible source of the cells of origin. They have also been used to help elucidate the molecular mechanisms of OS development. Several types of sarcomas have been modeled by transforming BM-MSCs with different oncogenic events (119, 120). Although the development of *in vivo* OS mouse models is essential for establishing more efficient and specific therapies for OS, the key question is how to create mouse models that can target the BM-MSCs that appear to be the OS cells of origin.

Most OS cases are sporadic in humans, and patients with OS frequently present with alterations in p53 (68, 121, 122). The hereditary p53 mutations associated with Li-Fraumeni syndrome also predispose patients to OS (123, 124). In mice, OS develops along with various tumors, mainly lymphomas, in both systemic *p53*-null and heterozygous individuals (125, 126). Thus, although the pathology of conventional *p53*-deficient mice indicates that p53 abnormalities contribute to OS development, these mice are not suitable for detailed analyses of the molecular mechanisms underlying osteosarcomagenesis. Therefore, mice with bone-associated cell lineage-specific *p53* gene alterations have been widely generated and used, as shown in **Table 1** (67–89). By crossing these mice with *p53*-deficient mice, the functions of other candidate oncogenes and anti-oncogenes in OS have been verified. *Rb* is a representative tumor suppressor gene candidate for OS pathogenesis. Patients with hereditary retinoblastoma have a significant predisposition for developing OS (127). However, the deficiency of Rb by itself does not cause OS, although it has been found to potentiate *p53*-deficient OS development (73, 74, 76). This shows the importance of the tumor-suppressive role of p53 in osteosarcomagenesis. Therefore, selecting cell lineage-specific *p53*-deficient mice that can effectively develop OS at a high rate may allow us to identify the cells of origin for OS and the molecular mechanism of OS development in these cells.

**Table 1:** Cell lineage-specific gene-targeting mouse studies for osteosarcoma.

Cre lines		Tumor suppressors	Oncogenes	References
***Osx/Sp7-ere (Osx/Sp 7-tTA)***		p53	Recql4	Ng et al. (67)
			Runx3/Myc	Otani et al. (68)
				Pourebrahim et al. (69)
		p53/Rb	E2Fs	Wu et al. (70)
			UHRF1	Wu et al. (71)
			Runx2	Lu et al. (72)
				Berman et al. (73)
				Calo et al. (74)
				Mutsaers et al. (75)
				Walkley et al. (76)
		p53/Wwox		Del Mare et al. (77)
		RanGAPI		Gong et al. (78)
			p53^R172H^/Ets2	Pourebrahim et al. (69)
			p53^R172H^/SBmut	Moriarity et al. (79)
			Wls/c-fos	Matsuoka et al. (80)
***Col1a1-cre***	2.3kb	p53	NICD	Tao et al. (81)
				Lin et al. (82)
		p53/Rb		Quist et al. (83)
		p53/Rb/Dlg		Shao et al. (84)
		p53^+/-^ (systemic)	JABl	Samsa et al. (85)
	3.6kb	p53		Lengner et al. (86)
***Prrxl-cre***		p53/Rb		Calo et al. (74)
				Lin et al. (82)
				Quist et al. (83)
			Recql4	Lu et al. (87)
***Ocn-cre***		p53/Ptch l		Chan et al. (88)
		p53/Rb		Quist et al. (83)
			SV40 T/t antigen	Molyneux et al. (89)
***Fgfr3-creER***		p53		Matsushita et al. (18)

The oncogenicity of *p53*-deficiency in early undifferentiated mesenchymal cells has been studied using a *Prrx1-cre* transgenic line. *Prrx1*-*cre*; *p53* ^fl/fl^ mice efficiently developed sarcomas, and OS accounted for approximately 60% of these tumors. The other 40% were tumors of other types, including rhabdomyosarcoma and undifferentiated sarcoma (74, 82) (**Figure 1B**). On the other hand, restricted *p53* deletion in cells committed to osteoblast or terminally differentiated osteoblast cells using *Col1a1*-*cre* or *Ocn*-*cre* lines, respectively, can cause OS in mice. However, not all individuals develop OS (82), and in the case of *Ocn*-*cre*, only about 40% of individuals develop OS even when Rb is deleted along with p53 (*Ocn*-*cre*; *p53* ^fl/fl^ *Rb* ^fl/fl^) (83) (**Figure 1B**). However, in osteoblast precursor-specific *p53*-deleted mice using *Osx*-*cre* (*Osx*-*cre*; *p53* ^fl/fl^), nearly 100% of the sarcomas that developed in most individuals were OS (68, 73, 74, 76), suggesting that the OS cells of origin are enriched in *Osx*-positive cells (**Figure 1B**). Recently, early onset of OS development was reported in mice with restrictive deletion of Ran GTPase-activating protein 1 (RanGAP1) using *Osx*-*cre*. However, no OS developed in cells using *Ocn*-*cre*, *Col1a1*-*cre*, periosteum-derived mesenchymal progenitor (PDMP)-specific *Ctsk*-*cre* chondrocyte-specific *Col2a1*-*cre*/*Col10a1*-*cre*, or *Prrx1*-*cre* (embryonic lethal with *Prrx1*-*cre*) (78). This observation also suggests that *Osx*-*cre* can target the cells of origin in OS or the cell population that contains them.

What makes *Osx*-positive osteoblast precursor cells/BM-MSCs tumorigenic when p53 is inactivated? The root of the tumorigenic process that occurs after the loss of p53 is the upregulation of Myc by Runx3, a Runx transcription factor (68) (**Figure 1B**). The oncogenicity of Runx2 in OS has been reported (128–130), but deletion of p53 markedly upregulates Runx3 rather than Runx2. The upregulation of Runx3 leads to dysregulation of Myc and the oncogenic Runx—Myc axis (68). This results in a malignant link between the loss of p53 and activation of Myc (131), the most potent oncogene in human cancer, including OS (132). The oncogenic Runx (Runx1)—Myc axis was also found to be essential for *p53*-deficient lymphomagenesis (133).

Thus, there are multiple OS mouse models using cell lineage-specific *cre* lines (134). However, all of them have been developed using conventional *cre* mouse lines, which do not allow for accurate cell-lineage tracing or make it possible to identify the genuine cells of origin for OS. In a recent study using *creER* mouse lines, Matsushita et al. found that *p53* deletion in *Fgfr3-creER* ^+^ endosteal stem cells, *Osx-creER* ^+^ osteoblast precursors, *Gli1-creER* ^+^ growth plate chondrocytes and metaphyseal mesenchymal progenitors, *Pthrp-creER* ^+^ growth plate stem cells in the resting zone, and *Lepr-cre* ^+^ marrow reticular stromal and their lineage cells showed distinct bone phenotypes. Among these models, *Fgfr3-creER*-expressing *p53*-deleted cells in young mice effectively generated OS, which broadly destroys pre-existing cortical bone from the endosteal marrow surface at or before 9 months of age (18). These results suggest that *Fgfr3* ^+^ cells contain the cells of origin for OS, as do *Osx* ^+^ cells (**Figure 1B**), and that novel *Fgfr3* ^+^ SSCs can provide a new perspective for the identification of the cells of origin for OS. They may also allow for the more precise redefinition of conventional BM-MSCs *in vivo*.

## Future challenges

In the past decade, we have acquired the powerful tool of scRNA-seq analysis, which can reveal the diversity of skeletal cells and predict their trajectories, as well as a mouse lineage-tracing approach for spatiotemporal validation. A comprehensive understanding of the dynamics of skeletal lineage cells has led to the elucidation of the biology of skeletal diseases, such as OS. The platforms and algorithms for scRNA-seq analysis are rapidly advancing. Although one of the disadvantages of scRNA-seq is the loss of spatial information, the latest single-cell spatial transcriptome analysis technique will accelerate research in all fields over the next decade (48, 135–140). Current studies using scRNA-seq have uncovered new skeletal cell types, which have received their own names. Understanding the actual cell clusters involved is becoming complicated because some of these computationally identified new cell types in distinct studies might overlap. Further communication between members of the research fields will be required for future investigations.

As of now, OS research has progressed exclusively through a series of analyses combining cell lineage-specific conventional *cre* mouse lines and floxed mice carrying cancer-related genes, mainly *p53*. However, the identification of the genuine cells of origin for OS is difficult because the conventional *cre* system continuously deletes target genes in a variety of cells, starting at the embryonic stage and continuing through all other life stages. Cancer research should focus on the *creER* line, which allows more precise control of recombination timing and cell type, in combination with fluorescent reporters and floxed lines of oncogenes and anti-oncogenes, to determine the cells of origin. This system can also provide a more precise molecular mechanism of tumorigenesis due to genomic alterations in a temporal and cell-specific manner and has already been implemented in other research fields involving major cancers. Therefore, it should be used more actively in future OS studies.

In this context, the mouse OS model generated from *Fgfr3-creER* ^+^ intraosseous stem cells lacking p53 is informative. Interestingly, OS is also caused by *p53* deletion in *Osx-creER^+^* cells located downstream of *Fgfr3-creER^+^* endosteal stem cells. In other words, multiple types of skeletal cells may behave as cells of origin for OS. The conventional *cre* system that has been used for OS research to date should be replaced by a more precisely controlled *creER* system, and future research should focus on clarifying cellular diversity and dynamics.

## Author contributions

Conception, design, and draft manuscript preparation: SO, MO, KI, and YM. All authors contributed to the article and approved the submitted version.

## References

[1] Matsushita Y Ono W Ono N. Skeletal stem cells for bone development and repair: diversity matters. Curr Osteoporos Rep (2020) 18(3):189–98. doi: 10.1007/s11914-020-00572-9 PMC7255932 32172443

[2] Morrison SJ Scadden DT. The bone marrow niche for haematopoietic stem cells. Nature. (2014) 505(7483):327–34. doi: 10.1038/nature12984 PMC4514480 24429631

[3] Greenbaum A Hsu YM Day RB Schuettpelz LG Christopher MJ Borgerding JN. CXCL12 in early mesenchymal progenitors is required for haematopoietic stem-cell maintenance. Nature. (2013) 495(7440):227–30. doi: 10.1038/nature11926 PMC3600148 23434756

[4] Arai F Hirao A Ohmura M Sato H Matsuoka S Takubo K. Tie2/angiopoietin-1 signaling regulates hematopoietic stem cell quiescence in the bone marrow niche. Cell. (2004) 118(2):149–61. doi: 10.1016/j.cell.2004.07.004 15260986

[5] Sacchetti B Funari A Michienzi S Di Cesare S Piersanti S Saggio I. Self-renewing osteoprogenitors in bone marrow sinusoids can organize a hematopoietic microenvironment. Cell. (2007) 131(2):324–36. doi: 10.1016/j.cell.2007.08.025 17956733

[6] Méndez-Ferrer S Bonnet D Steensma DP Hasserjian RP Ghobrial IM Gribben JG. Bone marrow niches in haematological malignancies. Nat Rev Canc (2020) 20(5):285–98. doi: 10.1038/s41568-020-0245-2 PMC9912977 32112045

[7] Asada N Kunisaki Y Pierce H Wang Z Fernandez NF Birbrair A. Differential cytokine contributions of perivascular haematopoietic stem cell niches. Nat Cell Biol (2017) 19(3):214–23. doi: 10.1038/ncb3475 PMC5467892 28218906

[8] Méndez-Ferrer S Michurina TV Ferraro F Mazloom AR Macarthur BD Lira SA. Mesenchymal and haematopoietic stem cells form a unique bone marrow niche. Nature. (2010) 466(7308):829–34. doi: 10.1038/nature09262 PMC3146551 20703299

[9] Omatsu Y Sugiyama T Kohara H Kondoh G Fujii N Kohno K. The essential functions of adipo-osteogenic progenitors as the hematopoietic stem and progenitor cell niche. Immunity. (2010) 33(3):387–99. doi: 10.1016/j.immuni.2010.08.017 20850355

[10] He S Nakada D Morrison SJ. Mechanisms of stem cell self-renewal. Annu Rev Cell Dev Biol (2009) 25:377–406. doi: 10.1146/annurev.cellbio.042308.113248 19575646

[11] Zakrzewski W Dobrzyński M Szymonowicz M Rybak Z. Stem cells: past, present, and future. Stem Cell Res Ther (2019) 10(1):68. doi: 10.1186/s13287-019-1165-5 30808416 PMC6390367

[12] Han Y Li X Zhang Y Chang F Ding J. Mesenchymal stem cells for regenerative medicine. Cells (2019) 8(8). doi: 10.3390/cells8080886 PMC6721852 31412678

[13] Pittenger MF Discher DE Péault BM Phinney DG Hare JM Caplan AI. Mesenchymal stem cell perspective: cell biology to clinical progress. NPJ Regener Med (2019) 4:22. doi: 10.1038/s41536-019-0083-6 PMC6889290 31815001

[14] Mizuhashi K Ono W Matsushita Y Sakagami N Takahashi A Saunders TL. Resting zone of the growth plate houses a unique class of skeletal stem cells. Nature. (2018) 563(7730):254–8. doi: 10.1038/s41586-018-0662-5 PMC6251707 30401834

[15] Debnath S Yallowitz AR McCormick J Lalani S Zhang T Xu R. Discovery of a periosteal stem cell mediating intramembranous bone formation. Nature. (2018) 562(7725):133–9. doi: 10.1038/s41586-018-0554-8 PMC6193396 30250253

[16] Zhou BO Yue R Murphy MM Peyer JG Morrison SJ. Leptin-receptor-expressing mesenchymal stromal cells represent the main source of bone formed by adult bone marrow. Cell Stem Cell (2014) 15(2):154–68. doi: 10.1016/j.stem.2014.06.008 PMC4127103 24953181

[17] Almeida M Kim HN Han L Zhou D Thostenson J Porter RM. Increased marrow adipogenesis does not contribute to age-dependent appendicular bone loss in female mice. Aging Cell (2020) 19(11):e13247. doi: 10.1111/acel.13247 33048436 PMC7681065

[18] Matsushita Y Liu J Chu AKY Tsutsumi-Arai C Nagata M Arai Y. Bone marrow endosteal stem cells dictate active osteogenesis and aggressive tumorigenesis. Nat Commun (2023) 14:2383. doi: 10.1038/s41467-023-38034-2 37185464 PMC10130060

[19] Bianco P. "Mesenchymal" stem cells. Annu Rev Cell Dev Biol (2014) 30:677–704. doi: 10.1146/annurev-cellbio-100913-013132 25150008

[20] Bianco P Robey PG. Skeletal stem cells. Development. (2015) 142(6):1023–7. doi: 10.1242/dev.102210 PMC4360182 25758217

[21] Friedenstein AJ Piatetzky-Shapiro II Petrakova KV. Osteogenesis in transplants of bone marrow cells. J Embryol Exp Morphol (1966) 16(3):381–90. doi: 10.1242/dev.16.3.381 5336210

[22] Mizoguchi T Ono N. The diverse origin of bone-forming osteoblasts. J Bone Miner Res (2021) 36(8):1432–47. doi: 10.1002/jbmr.4410 PMC8338797 34213032

[23] Perrin S Colnot C. Periosteal skeletal stem and progenitor cells in bone regeneration. Curr Osteoporos Rep (2022) 20(5):334–43. doi: 10.1007/s11914-022-00737-8 35829950

[24] Kurenkova AD Medvedeva EV Newton PT Chagin AS. Niches for skeletal stem cells of mesenchymal origin. Front Cell Dev Biol (2020) 8:592. doi: 10.3389/fcell.2020.00592 32754592 PMC7366157

[25] Mancinelli L Intini G. Age-associated declining of the regeneration potential of skeletal stem/progenitor cells. Front Physiol (2023) 14:1087254. doi: 10.3389/fphys.2023.1087254 36818437 PMC9931727

[26] Chan CK Seo EY Chen JY Lo D McArdle A Sinha R. Identification and specification of the mouse skeletal stem cell. Cell. (2015) 160(1-2):285–98. doi: 10.1016/j.cell.2014.12.002 PMC4297645 25594184

[27] Morikawa S Mabuchi Y Kubota Y Nagai Y Niibe K Hiratsu E. Prospective identification, isolation, and systemic transplantation of multipotent mesenchymal stem cells in murine bone marrow. J Exp Med (2009) 206(11):2483–96. doi: 10.1084/jem.20091046 PMC2768869 19841085

[28] Breitbach M Kimura K Luis TC Fuegemann CJ Woll PS Hesse M. *In Vivo* Labeling by CD73 marks multipotent stromal cells and highlights endothelial heterogeneity in the bone marrow niche. Cell Stem Cell (2018) 22(2):262–76.e7. doi: 10.1016/j.stem.2018.01.008 29451855

[29] Chan CKF Gulati GS Sinha R Tompkins JV Lopez M Carter AC. Identification of the human skeletal stem cell. Cell. (2018) 175(1):43–56.e21. doi: 10.1016/j.cell.2018.07.029 30241615 PMC6400492

[30] Das B Kashino SS Pulu I Kalita D Swami V Yeger H. CD271(+) bone marrow mesenchymal stem cells may provide a niche for dormant mycobacterium tuberculosis. Sci Transl Med (2013) 5(170):170ra13. doi: 10.1126/scitranslmed.3004912 PMC3616630 23363977

[31] Yang ZX Han ZB Ji YR Wang YW Liang L Chi Y. CD106 identifies a subpopulation of mesenchymal stem cells with unique immunomodulatory properties. PloS One (2013) 8(3):e59354. doi: 10.1371/journal.pone.0059354 23555021 PMC3595282

[32] Kretzschmar K Watt FM. Lineage tracing. Cell (2012) 148(1-2):33–45. doi: 10.1016/j.cell.2012.01.002 22265400

[33] Couasnay G Madel MB Lim J Lee B Elefteriou F. Sites of cre-recombinase activity in mouse lines targeting skeletal cells. J Bone Miner Res (2021) 36(9):1661–79. doi: 10.1002/jbmr.4415 34278610

[34] Matsushita Y Ono W Ono N. Growth plate skeletal stem cells and their transition from cartilage to bone. Bone. (2020) 136:115359. doi: 10.1016/j.bone.2020.115359 32276155 PMC7246136

[35] Tikhonova AN Dolgalev I Hu H Sivaraj KK Hoxha E Cuesta-Domínguez ÁChecktae. The bone marrow microenvironment at single-cell resolution. Nature. (2019) 569(7755):222–8. doi: 10.1038/s41586-019-1104-8 PMC6607432 30971824

[36] Kansara M Teng MW Smyth MJ Thomas DM. Translational biology of osteosarcoma. Nat Rev Canc (2014) 14(11):722–35. doi: 10.1038/nrc3838 25319867

[37] Choi JH Ro JY. The 2020 WHO classification of tumors of bone: an updated review. Adv Anat Pathol (2021) 28(3):119–38. doi: 10.1097/PAP.0000000000000293 33480599

[38] Feil R Brocard J Mascrez B LeMeur M Metzger D Chambon P. Ligand-activated site-specific recombination in mice. Proc Natl Acad Sci U S A (1996) 93(20):10887–90. doi: 10.1073/pnas.93.20.10887 PMC38252 8855277

[39] Shimshek DR Kim J Hübner MR Spergel DJ Buchholz F Casanova E. Codon-improved cre recombinase (iCre) expression in the mouse. Genesis. (2002) 32(1):19–26. doi: 10.1002/gene.10023 11835670

[40] Madisen L Zwingman TA Sunkin SM Oh SW Zariwala HA Gu H. A robust and high-throughput cre reporting and characterization system for the whole mouse brain. Nat Neurosci (2010) 13(1):133–40. doi: 10.1038/nn.2467 PMC2840225 20023653

[41] He M Tucciarone J Lee S Nigro MJ Kim Y Levine JM. Strategies and tools for combinatorial targeting of GABAergic neurons in mouse cerebral cortex. Neuron. (2016) 91(6):1228–43. doi: 10.1016/j.neuron.2016.08.021 PMC5223593 27618674

[42] Snippert HJ van der Flier LG Sato T van Es JH van den Born M Kroon-Veenboer C. Intestinal crypt homeostasis results from neutral competition between symmetrically dividing Lgr5 stem cells. Cell. (2010) 143(1):134–44. doi: 10.1016/j.cell.2010.09.016 20887898

[43] Ye W Lian Q Ye C Wu X. A survey on methods for predicting polyadenylation sites from DNA sequences, bulk RNA-seq, and single-cell RNA-seq. Genomics Proteomics Bioinf (2022). doi: 10.1016/j.gpb.2022.09.005 PMC10372920 36167284

[44] Kulkarni A Anderson AG Merullo DP Konopka G. Beyond bulk: a review of single cell transcriptomics methodologies and applications. Curr Opin Biotechnol (2019) 58:129–36. doi: 10.1016/j.copbio.2019.03.001 PMC6710112 30978643

[45] Stoeckius M Hafemeister C Stephenson W Houck-Loomis B Chattopadhyay PK Swerdlow H. Simultaneous epitope and transcriptome measurement in single cells. Nat Methods (2017) 14(9):865–8. doi: 10.1038/nmeth.4380 PMC5669064 28759029

[46] Cusanovich DA Hill AJ Aghamirzaie D Daza RM Pliner HA Berletch JB. A single-cell atlas of *In Vivo* mammalian chromatin accessibility. Cell. (2018) 174(5):1309–1324.e18. doi: 10.1016/j.cell.2018.06.052 30078704 PMC6158300

[47] Stuart T Butler A Hoffman P Hafemeister C Papalexi E Mauck WM. Comprehensive integration of single-cell data. Cell. (2019) 177(7):1888–1902.e21. doi: 10.1016/j.cell.2019.05.031 31178118 PMC6687398

[48] Rodriques SG Stickels RR Goeva A Martin CA Murray E Vanderburg CR. Slide-seq: a scalable technology for measuring genome-wide expression at high spatial resolution. Science. (2019) 363(6434):1463–7. doi: 10.1126/science.aaw1219 PMC6927209 30923225

[49] Hao Y Hao S Andersen-Nissen E Mauck WM Zheng S Butler A. Integrated analysis of multimodal single-cell data. Cell. (2021) 184(13):3573–87. doi: 10.1016/j.cell.2021.04.048 PMC8238499 34062119

[50] Matsushita Y Ono W Ono N. Synergy of single-cell sequencing analyses and. Biocell. (2022) 46(5):1157–62. doi: 10.32604/biocell.2022.018960 PMC9037299 35475293

[51] Krebsbach PH Kuznetsov SA Bianco P Robey PG. Bone marrow stromal cells: characterization and clinical application. Crit Rev Oral Biol Med (1999) 10(2):165–81. doi: 10.1177/10454411990100020401 10759420

[52] Pittenger MF Mackay AM Beck SC Jaiswal RK Douglas R Mosca JD. Multilineage potential of adult human mesenchymal stem cells. Science. (1999) 284(5411):143–7. doi: 10.1126/science.284.5411.143 10102814

[53] Zhong L Yao L Tower RJ Wei Y Miao Z Park J. Single cell transcriptomics identifies a unique adipose lineage cell population that regulates bone marrow environment. Elife. (2020) 04 14:9. doi: 10.7554/eLife.54695 PMC7220380 32286228

[54] Wolock SL Krishnan I Tenen DE Matkins V Camacho V Patel S. Mapping distinct bone marrow niche populations and their differentiation paths. Cell Rep (2019) 28(2):302–311.e5. doi: 10.1016/j.celrep.2019.06.031 31291568 PMC6684313

[55] Xu C Dinh VV Kruse K Jeong HW Watson EC Adams S. Induction of osteogenesis by bone-targeted notch activation. Elife (2022) 11:e60183. doi: 10.7554/eLife.60183 35119364 PMC8880996

[56] Sivaraj KK Jeong HW Dharmalingam B Zeuschner D Adams S Potente M. Regional specialization and fate specification of bone stromal cells in skeletal development. Cell Rep (2021) 36(2):109352. doi: 10.1016/j.celrep.2021.109352 34260921 PMC8293626

[57] Mo C Guo J Qin J Zhang X Sun Y Wei H. Single-cell transcriptomics of LepR-positive skeletal cells reveals heterogeneous stress-dependent stem and progenitor pools. EMBO J (2022) 41(4):e108415. doi: 10.15252/embj.2021108415 34957577 PMC8844986

[58] Ambrosi TH Marecic O McArdle A Sinha R Gulati GS Tong X. Aged skeletal stem cells generate an inflammatory degenerative niche. Nature. (2021) 597(7875):256–62. doi: 10.1038/s41586-021-03795-7 PMC8721524 34381212

[59] Baryawno N Przybylski D Kowalczyk MS Kfoury Y Severe N Gustafsson K. A cellular taxonomy of the bone marrow stroma in homeostasis and leukemia. Cell. (2019) 177(7):1915–1932.e16. doi: 10.1016/j.cell.2019.04.040 31130381 PMC6570562

[60] Baccin C Al-Sabah J Velten L Helbling PM Grünschläger F Hernández-Malmierca P. Combined single-cell and spatial transcriptomics reveal the molecular, cellular and spatial bone marrow niche organization. Nat Cell Biol (2020) 22(1):38–48. doi: 10.1038/s41556-019-0439-6 31871321 PMC7610809

[61] Matsushita Y Nagata M Kozloff KM Welch JD Mizuhashi K Tokavanich N. A wnt-mediated transformation of the bone marrow stromal cell identity orchestrates skeletal regeneration. Nat Commun (2020) 11(1):332. doi: 10.1038/s41467-019-14029-w 31949165 PMC6965122

[62] Matsushita Y Chu AKY Ono W Welch JD Ono N. Intercellular interactions of an adipogenic CXCL12-expressing stromal cell subset in murine bone marrow. J Bone Miner Res (2021) 36(6):1145–58. doi: 10.1002/jbmr.4282 PMC8605623 33651379

[63] Trapnell C Cacchiarelli D Grimsby J Pokharel P Li S Morse M. The dynamics and regulators of cell fate decisions are revealed by pseudotemporal ordering of single cells. Nat Biotechnol (2014) 32(4):381–6. doi: 10.1038/nbt.2859 PMC4122333 24658644

[64] Qiu X Hill A Packer J Lin D Ma YA Trapnell C. Single-cell mRNA quantification and differential analysis with census. Nat Methods (2017) 14(3):309–15. doi: 10.1038/nmeth.4150 PMC5330805 28114287

[65] La Manno G Soldatov R Zeisel A Braun E Hochgerner H Petukhov V. RNA Velocity of single cells. Nature. (2018) 560(7719):494–8. doi: 10.1038/s41586-018-0414-6 PMC6130801 30089906

[66] Bergen V Lange M Peidli S Wolf FA Theis FJ. Generalizing RNA velocity to transient cell states through dynamical modeling. Nat Biotechnol (2020) 38(12):1408–14. doi: 10.1038/s41587-020-0591-3 32747759

[67] Ng AJ Walia MK Smeets MF Mutsaers AJ Sims NA Purton LE. The DNA helicase recql4 is required for normal osteoblast expansion and osteosarcoma formation. PloS Genet (2015) 11(4):e1005160. doi: 10.1371/journal.pgen.1005160 25859855 PMC4393104

[68] Otani S Date Y Ueno T Ito T Kajikawa S Omori K. Runx3 is required for oncogenic myc upregulation in p53-deficient osteosarcoma. Oncogene. (2022) 41(5):683–91. doi: 10.1038/s41388-021-02120-w 34803166

[69] Pourebrahim R Zhang Y Liu B Gao R Xiong S Lin PP. Integrative genome analysis of somatic p53 mutant osteosarcomas identifies Ets2-dependent regulation of small nucleolar RNAs by mutant p53 protein. Genes Dev (2017) 31(18):1847–57. doi: 10.1101/gad.304972.117 PMC5695086 29021240

[70] Wu SC Benavente CA. Chromatin remodeling protein HELLS is upregulated by inactivation of the RB-E2F pathway and is nonessential for osteosarcoma tumorigenesis. Oncotarget. (2018) 9(66):32580–92. doi: 10.18632/oncotarget.25953 PMC6135688 30220967

[71] Wu SC Kim A Gu Y Martinez DI Zocchi L Chen CC. UHRF1 overexpression promotes osteosarcoma metastasis through altered exosome production and AMPK/SEMA3E suppression. Oncogenesis. (2022) 11(1):51. doi: 10.1038/s41389-022-00430-6 36068209 PMC9448786

[72] Lu Y Gitelis S Lei G Ding M Maki C Mira RR. Research findings working with the p53 and Rb1 targeted osteosarcoma mouse model. Am J Cancer Res (2014) 4(3):234–44. PMC4065404 24959378

[73] Berman SD Calo E Landman AS Danielian PS Miller ES West JC. Metastatic osteosarcoma induced by inactivation of Rb and p53 in the osteoblast lineage. Proc Natl Acad Sci U S A (2008) 105(33):11851–6. doi: 10.1073/pnas.0805462105 PMC2575280 18697945

[74] Calo E Quintero-Estades JA Danielian PS Nedelcu S Berman SD Lees JA. Rb Regulates fate choice and lineage commitment in vivo. Nature. (2010) 466(7310):1110–4. doi: 10.1038/nature09264 PMC2933655 20686481

[75] Mutsaers AJ Ng AJ Baker EK Russell MR Chalk AM Wall M. Modeling distinct osteosarcoma subtypes *in vivo* using cre:lox and lineage-restricted transgenic shRNA. Bone. (2013) 55(1):166–78. doi: 10.1016/j.bone.2013.02.016 23486187

[76] Walkley CR Qudsi R Sankaran VG Perry JA Gostissa M Roth SI. Conditional mouse osteosarcoma, dependent on p53 loss and potentiated by loss of Rb, mimics the human disease. Genes Dev (2008) 22(12):1662–76. doi: 10.1101/gad.1656808 PMC2428063 18559481

[77] Del Mare S Husanie H Iancu O Abu-Odeh M Evangelou K Lovat F. WWOX and p53 dysregulation synergize to drive the development of osteosarcoma. Cancer Res (2016) 76(20):6107–17. doi: 10.1158/0008-5472.CAN-16-0621 PMC5146760 27550453

[78] Gong Y Zou S Deng D Wang L Hu H Qiu Z. Loss of RanGAP1 drives chromosome instability and rapid tumorigenesis of osteosarcoma. Dev Cell (2023) 58(3):192–210.e11. doi: 10.1016/j.devcel.2022.12.012 36696903

[79] Moriarity BS Otto GM Rahrmann EP Rathe SK Wolf NK Weg MT. A sleeping beauty forward genetic screen identifies new genes and pathways driving osteosarcoma development and metastasis. Nat Genet (2015) 47(6):615–24. doi: 10.1038/ng.3293 PMC4767150 25961939

[80] Matsuoka K Bakiri L Wolff LI Linder M Mikels-Vigdal A Patiño-García A. Wnt signaling and Loxl2 promote aggressive osteosarcoma. Cell Res (2020) 30(10):885–901. doi: 10.1038/s41422-020-0370-1 32686768 PMC7608146

[81] Tao J Jiang MM Jiang L Salvo JS Zeng HC Dawson B. Notch activation as a driver of osteogenic sarcoma. Cancer Cell (2014) 26(3):390–401. doi: 10.1016/j.ccr.2014.07.023 25203324 PMC4159617

[82] Lin PP Pandey MK Jin F Raymond AK Akiyama H Lozano G. Targeted mutation of p53 and Rb in mesenchymal cells of the limb bud produces sarcomas in mice. Carcinogenesis. (2009) 30(10):1789–95. doi: 10.1093/carcin/bgp180 PMC4141195 19635748

[83] Quist T Jin H Zhu JF Smith-Fry K Capecchi MR Jones KB. The impact of osteoblastic differentiation on osteosarcomagenesis in the mouse. Oncogene. (2015) 34(32):4278–84. doi: 10.1038/onc.2014.354 PMC4411188 25347737

[84] Shao YW Wood GA Lu J Tang QL Liu J Molyneux S. Cross-species genomics identifies DLG2 as a tumor suppressor in osteosarcoma. Oncogene. (2019) 38(2):291–8. doi: 10.1038/s41388-018-0444-4 PMC6756098 30093633

[85] Samsa WE Mamidi MK Bashur LA Elliott R Miron A Chen Y. The crucial p53-dependent oncogenic role of JAB1 in osteosarcoma in vivo. Oncogene. (2020) 39(23):4581–91. doi: 10.1038/s41388-020-1320-6 PMC7274902 32390003

[86] Lengner CJ Steinman HA Gagnon J Smith TW Henderson JE Kream BE. Osteoblast differentiation and skeletal development are regulated by Mdm2-p53 signaling. J Cell Biol (2006) 172(6):909–21. doi: 10.1083/jcb.200508130 PMC2063734 16533949

[87] Lu L Harutyunyan K Jin W Wu J Yang T Chen Y. RECQL4 regulates p53 function *In Vivo* during skeletogenesis. J Bone Miner Res (2015) 30(6):1077–89. doi: 10.1002/jbmr.2436 25556649

[88] Chan LH Wang W Yeung W Deng Y Yuan P Mak KK. Hedgehog signaling induces osteosarcoma development through Yap1 and H19 overexpression. Oncogene. (2014) 33(40):4857–66. doi: 10.1038/onc.2013.433 24141783

[89] Molyneux SD Di Grappa MA Beristain AG McKee TD Wai DH Paderova J. Prkar1a is an osteosarcoma tumor suppressor that defines a molecular subclass in mice. J Clin Invest (2010) 120(9):3310–25. doi: 10.1172/JCI42391 PMC2929719 20697156

[90] Martin JF Olson EN. Identification of a prx1 limb enhancer. Genesis. (2000) 26(4):225–9. doi: 10.1002/(SICI)1526-968X(200004)26:4<225::AID-GENE10>3.0.CO;2-F 10748458

[91] Logan M Martin JF Nagy A Lobe C Olson EN Tabin CJ. Expression of cre recombinase in the developing mouse limb bud driven by a prxl enhancer. Genesis. (2002) 33(2):77–80. doi: 10.1002/gene.10092 12112875

[92] Akiyama H Kim JE Nakashima K Balmes G Iwai N Deng JM. Osteo-chondroprogenitor cells are derived from Sox9 expressing precursors. Proc Natl Acad Sci U S A (2005) 102(41):14665–70. doi: 10.1073/pnas.0504750102 PMC1239942 16203988

[93] Soeda T Deng JM de Crombrugghe B Behringer RR Nakamura T Akiyama H. Sox9-expressing precursors are the cellular origin of the cruciate ligament of the knee joint and the limb tendons. Genesis. (2010) 48(11):635–44. doi: 10.1002/dvg.20667 PMC3982414 20806356

[94] Ono N Ono W Nagasawa T Kronenberg HM. A subset of chondrogenic cells provides early mesenchymal progenitors in growing bones. Nat Cell Biol (2014) 16(12):1157–67. doi: 10.1038/ncb3067 PMC4250334 25419849

[95] Shi Y He G Lee WC McKenzie JA Silva MJ Long F. Gli1 identifies osteogenic progenitors for bone formation and fracture repair. Nat Commun (2017) 8(1):2043. doi: 10.1038/s41467-017-02171-2 29230039 PMC5725597

[96] Yang L Tsang KY Tang HC Chan D Cheah KS. Hypertrophic chondrocytes can become osteoblasts and osteocytes in endochondral bone formation. Proc Natl Acad Sci U S A (2014) 111(33):12097–102. doi: 10.1073/pnas.1302703111 PMC4143064 25092332

[97] Long JT Leinroth A Liao Y Ren Y Mirando AJ Nguyen T. Hypertrophic chondrocytes serve as a reservoir for marrow-associated skeletal stem and progenitor cells, osteoblasts, and adipocytes during skeletal development. Elife (2022) 18:11. doi: 10.7554/eLife.76932 PMC8893718 35179487

[98] Matsushita Y Chu AKY Tsutsumi-Arai C Orikasa S Nagata M Wong SY. The fate of early perichondrial cells in developing bones. Nat Commun (2022) 13(1):7319. doi: 10.1038/s41467-022-34804-6 36443296 PMC9705540

[99] Maes C Kobayashi T Selig MK Torrekens S Roth SI Mackem S. Osteoblast precursors, but not mature osteoblasts, move into developing and fractured bones along with invading blood vessels. Dev Cell (2010) 19(2):329–44. doi: 10.1016/j.devcel.2010.07.010 PMC3540406 20708594

[100] Mizoguchi T Pinho S Ahmed J Kunisaki Y Hanoun M Mendelson A. Osterix marks distinct waves of primitive and definitive stromal progenitors during bone marrow development. Dev Cell (2014) 29(3):340–9. doi: 10.1016/j.devcel.2014.03.013 PMC4051418 24823377

[101] Tsukasaki M Komatsu N Negishi-Koga T Huynh NC Muro R Ando Y. Periosteal stem cells control growth plate stem cells during postnatal skeletal growth. Nat Commun (2022) 13(1):4166. doi: 10.1038/s41467-022-31592-x 35851381 PMC9293991

[102] Seike M Omatsu Y Watanabe H Kondoh G Nagasawa T. Stem cell niche-specific Ebf3 maintains the bone marrow cavity. Genes Dev (2018) 32(5-6):359–72. doi: 10.1101/gad.311068.117 PMC5900710 29563184

[103] Worthley DL Churchill M Compton JT Tailor Y Rao M Si Y. Gremlin 1 identifies a skeletal stem cell with bone, cartilage, and reticular stromal potential. Cell. (2015) 160(1-2):269–84. doi: 10.1016/j.cell.2014.11.042 PMC4436082 25594183

[104] Nakashima K Zhou X Kunkel G Zhang Z Deng JM Behringer RR. The novel zinc finger-containing transcription factor osterix is required for osteoblast differentiation and bone formation. Cell. (2002) 108(1):17–29. doi: 10.1016/s0092-8674(01)00622-5 11792318

[105] Hojo H Ohba S He X Lai LP McMahon AP. Sp7/Osterix is restricted to bone-forming vertebrates where it acts as a dlx Co-factor in osteoblast specification. Dev Cell (2016) 37(3):238–53. doi: 10.1016/j.devcel.2016.04.002 PMC4964983 27134141

[106] Liu Y Strecker S Wang L Kronenberg MS Wang W Rowe DW. Osterix-cre labeled progenitor cells contribute to the formation and maintenance of the bone marrow stroma. PloS One (2013) 8(8):e71318. doi: 10.1371/journal.pone.0071318 23951132 PMC3738599

[107] Böhm AM Dirckx N Tower RJ Peredo N Vanuytven S Theunis K. Activation of skeletal stem and progenitor cells for bone regeneration is driven by PDGFRβ signaling. Dev Cell (2019) 51(2):236–254.e12. doi: 10.1016/j.devcel.2019.08.013 31543445

[108] Chen J Shi Y Regan J Karuppaiah K Ornitz DM Long F. Osx-cre targets multiple cell types besides osteoblast lineage in postnatal mice. PloS One (2014) 9(1):e85161. doi: 10.1371/journal.pone.0085161 24454809 PMC3893188

[109] Zhong Z Zylstra-Diegel CR Schumacher CA Baker JJ Carpenter AC Rao S. Wntless functions in mature osteoblasts to regulate bone mass. Proc Natl Acad Sci U S A (2012) 109(33):E2197–204. doi: 10.1073/pnas.1120407109 PMC3421196 22745162

[110] Xiao Z Huang J Cao L Liang Y Han X Quarles LD. Osteocyte-specific deletion of Fgfr1 suppresses FGF23. PloS One (2014) 9(8):e104154. doi: 10.1371/journal.pone.0104154 25089825 PMC4121311

[111] Zhang J Link DC. Targeting of mesenchymal stromal cells by cre-recombinase transgenes commonly used to target osteoblast lineage cells. J Bone Miner Res (2016) 31(11):2001–7. doi: 10.1002/jbmr.2877 PMC5523961 27237054

[112] Lim J Burclaff J He G Mills JC Long F. Unintended targeting of. Bone Res (2017) 5:16049. doi: 10.1038/boneres.2016.49 28163952 PMC5282469

[113] Matic I Matthews BG Wang X Dyment NA Worthley DL Rowe DW. Quiescent bone lining cells are a major source of osteoblasts during adulthood. Stem Cells (2016) 34(12):2930–42. doi: 10.1002/stem.2474 PMC5450652 27507737

[114] Frey JL Kim SP Li Z Wolfgang MJ Riddle RC. β-catenin directs long-chain fatty acid catabolism in the osteoblasts of Male mice. Endocrinology. (2018) 159(1):272–84. doi: 10.1210/en.2017-00850 PMC5761587 29077850

[115] Liu F Woitge HW Braut A Kronenberg MS Lichtler AC Mina M. Expression and activity of osteoblast-targeted cre recombinase transgenes in murine skeletal tissues. Int J Dev Biol (2004) 48(7):645–53. doi: 10.1387/ijdb.041816fl 15470637

[116] Dacquin R Starbuck M Schinke T Karsenty G. Mouse alpha1(I)-collagen promoter is the best known promoter to drive efficient cre recombinase expression in osteoblast. Dev Dyn (2002) 224(2):245–51. doi: 10.1002/dvdy.10100 12112477

[117] Papaioannou G Mirzamohammadi F Kobayashi T. Ras signaling regulates osteoprogenitor cell proliferation and bone formation. Cell Death Dis (2016) 7(10):e2405. doi: 10.1038/cddis.2016.314 27735946 PMC5133981

[118] García-Castro J Trigueros C Madrenas J Pérez-Simón JA Rodriguez R Menendez P. Mesenchymal stem cells and their use as cell replacement therapy and disease modelling tool. J Cell Mol Med (2008) 12(6B):2552–65. doi: 10.1111/j.1582-4934.2008.00516.x PMC3828873 19210755

[119] Rodríguez R García-Castro J Trigueros C García Arranz M Menéndez P. Multipotent mesenchymal stromal cells: clinical applications and cancer modeling. Adv Exp Med Biol (2012) 741:187–205. doi: 10.1007/978-1-4614-2098-9_13 22457111

[120] Rubio R Gutierrez-Aranda I Sáez-Castillo AI Labarga A Rosu-Myles M Gonzalez-Garcia S. The differentiation stage of p53-rb-deficient bone marrow mesenchymal stem cells imposes the phenotype of *in vivo* sarcoma development. Oncogene. (2013) 32(41):4970–80. doi: 10.1038/onc.2012.507 23222711

[121] Miller CW Aslo A Tsay C Slamon D Ishizaki K Toguchida J. Frequency and structure of p53 rearrangements in human osteosarcoma. Cancer Res (1990) 50(24):7950–4. 2253237

[122] Chen X Bahrami A Pappo A Easton J Dalton J Hedlund E. Recurrent somatic structural variations contribute to tumorigenesis in pediatric osteosarcoma. Cell Rep (2014) 7(1):104–12. doi: 10.1016/j.celrep.2014.03.003 PMC4096827 24703847

[123] Porter DE Holden ST Steel CM Cohen BB Wallace MR Reid R. A significant proportion of patients with osteosarcoma may belong to Li-fraumeni cancer families. J Bone Joint Surg Br (1992) 74(6):883–6. doi: 10.1302/0301-620X.74B6.1447251 1447251

[124] Bougeard G Renaux-Petel M Flaman JM Charbonnier C Fermey P Belotti M. Revisiting Li-fraumeni syndrome from TP53 mutation carriers. J Clin Oncol (2015) 33(21):2345–52. doi: 10.1200/JCO.2014.59.5728 26014290

[125] Donehower LA Harvey M Slagle BL McArthur MJ Montgomery CA Butel JS. Mice deficient for p53 are developmentally normal but susceptible to spontaneous tumours. Nature. (1992) 356(6366):215–21. doi: 10.1038/356215a0 1552940

[126] Jacks T Remington L Williams BO Schmitt EM Halachmi S Bronson RT. Tumor spectrum analysis in p53-mutant mice. Curr Biol (1994) 4(1):1–7. doi: 10.1016/s0960-9822(00)00002-6 7922305

[127] Gurney JG Severson RK Davis S Robison LL. Incidence of cancer in children in the united states. Sex- race- 1-year age-specific rates by histologic type Canc (1995) 75(8):2186–95. doi: 10.1002/1097-0142(19950415)75:8<2186::aid-cncr2820750825>3.0.co;2-f 7697611

[128] Martin JW Zielenska M Stein GS van Wijnen AJ Squire JA. The role of RUNX2 in osteosarcoma oncogenesis. Sarcoma. (2011) 2011:282745. doi: 10.1155/2011/282745 21197465 PMC3005824

[129] Ito Y Bae SC Chuang LS. The RUNX family: developmental regulators in cancer. Nat Rev Canc (2015) 15(2):81–95. doi: 10.1038/nrc3877 25592647

[130] Shin MH He Y Marrogi E Piperdi S Ren L Khanna C. A RUNX2-mediated epigenetic regulation of the survival of p53 defective cancer cells. PloS Genet (2016) 12(2):e1005884. doi: 10.1371/journal.pgen.1005884 26925584 PMC4771715

[131] Ulz P Heitzer E Speicher MR. Co-Occurrence of MYC amplification and TP53 mutations in human cancer. Nat Genet (2016) 48(2):104–6. doi: 10.1038/ng.3468 26813759

[132] Jain M Arvanitis C Chu K Dewey W Leonhardt E Trinh M. Sustained loss of a neoplastic phenotype by brief inactivation of MYC. Science. (2002) 297(5578):102–4. doi: 10.1126/science.1071489 12098700

[133] Date Y Taniuchi I Ito K. Oncogenic Runx1-myc axis in p53-deficient thymic lymphoma. Gene. (2022) 819:146234. doi: 10.1016/j.gene.2022.146234 35114276

[134] Walia MK Castillo-Tandazo W Mutsaers AJ Martin TJ Walkley CR. Murine models of osteosarcoma: a piece of the translational puzzle. J Cell Biochem (2018) 119(6):4241–50. doi: 10.1002/jcb.26601 29236321

[135] Ståhl PL Salmén F Vickovic S Lundmark A Navarro JF Magnusson J. Visualization and analysis of gene expression in tissue sections by spatial transcriptomics. Science. (2016) 353(6294):78–82. doi: 10.1126/science.aaf2403 27365449

[136] Liu Y Yang M Deng Y Su G Enninful A Guo CC. High-Spatial-Resolution multi-omics sequencing *via* deterministic barcoding in tissue. Cell. (2020) 183(6):1665–1681.e18. doi: 10.1016/j.cell.2020.10.026 33188776 PMC7736559

[137] Cho CS Xi J Si Y Park SR Hsu JE Kim M. Microscopic examination of spatial transcriptome using seq-scope. Cell. (2021) 184(13):3559–3572.e22. doi: 10.1016/j.cell.2021.05.010 34115981 PMC8238917

[138] Deng Y Bartosovic M Kukanja P Zhang D Liu Y Su G. Spatial-CUT&Tag: spatially resolved chromatin modification profiling at the cellular level. Science (2022) 375(6581):681–6. doi: 10.1126/science.abg7216 PMC7612972 35143307

[139] Chen A Liao S Cheng M Ma K Wu L Lai Y. Spatiotemporal transcriptomic atlas of mouse organogenesis using DNA nanoball-patterned arrays. Cell. (2022) 185(10):1777–92.e21. doi: 10.1016/j.cell.2022.04.003 35512705

[140] Seferbekova Z Lomakin A Yates LR Gerstung M. Spatial biology of cancer evolution. Nat Rev Genet (2022) 24(5):295–313. doi: 10.1038/s41576-022-00553-x 36494509

